# An organic transistor-based system for reference-less electrophysiological monitoring of excitable cells

**DOI:** 10.1038/srep08807

**Published:** 2015-03-06

**Authors:** A. Spanu, S. Lai, P. Cosseddu, M. Tedesco, S. Martinoia, A. Bonfiglio

**Affiliations:** 1 University of Cagliari – Dept. of Electrical and Electronic Engineering, Piazza d'Armi, 09123 Cagliari, Italy; 2 University of Genova – Dept. of Informatics, Bioengineering, Robotics and System Engineering, Via Opera Pia 13, 16145 Genova, Italy; 3 CNR – Institute of Nanoscience, Via Campi 213/A, 41125 Modena, Italy

## Abstract

In the last four decades, substantial advances have been done in the understanding of the electrical behavior of excitable cells. From the introduction in the early 70's of the Ion Sensitive Field Effect Transistor (ISFET), a lot of effort has been put in the development of more and more performing transistor-based devices to reliably interface electrogenic cells such as, for example, cardiac myocytes and neurons. However, depending on the type of application, the electronic devices used to this aim face several problems like the intrinsic rigidity of the materials (associated with foreign body rejection reactions), lack of transparency and the presence of a reference electrode. Here, an innovative system based on a novel kind of organic thin film transistor (OTFT), called organic charge modulated FET (OCMFET), is proposed as a flexible, transparent, reference-less transducer of the electrical activity of electrogenic cells. The exploitation of organic electronics in interfacing the living matters will open up new perspectives in the electrophysiological field allowing us to head toward a modern era of flexible, reference-less, and low cost probes with high-spatial and high-temporal resolution for a new generation of in-vitro and in-vivo monitoring platforms.

The effective connection between cellular tissues and technological platforms is crucial for the success of many bionic applications as neural prostheses and hybrids for studying information processing in neuronal networks. Moreover, the recent attention for disposable, low-cost and reliable cell-to-chip interface systems for high-throughput in-vitro toxicity assays and pharmacology is becoming an urgent request because of the new international regulatory test guidelines (both in Europe and USA). Therefore, engineering the so-called bio-electronic interface is the subject of many technological and basic/applied researches. Specifically related to the cell (neuronal)-electronic interface, two different kinds of devices have been extensively used over the past thirty years, namely microelectrodes arrays (MEAs)^1^,^2^ and field effect devices^3^,^4^,^5^,^6^. More recently, organic semiconductors have attracted a considerable interest in this field^7^,^8^,^9^ because they have the potential to fulfill many critical requirements for biomedical and biotechnological applications such as biological compatibility, mechanical flexibility, and optical transparency. Moreover, devices based on organic semiconductors can be fabricated on flexible low-cost plastic substrates, with micrometric resolution, over large areas, and using cost-efficient technologies. All these features could allow addressing a wide variety of novel applications ranging from *in-vitro* to *in-vivo* cell biology and addressing unsolved problems of mechanical adaptability (e.g., high density flexible transducers on catheters) or of multi-parameters analysis on a micro-scale (e.g., disposable and sensorized smart-petri dish). So far, in addition to passive electrodes made of organic conductive polymers^10^, organic electrochemical transistors (OECTs) have been mainly proposed^11^,^12^ because of their ability to conduct ionic and electronic charges and to be operated in liquid at very low voltages, which represents a crucial requirement in presence of living cells or tissues. Organic Thin Film Transistors (OTFTs) have not yet been employed so far to this aim for two main reasons: 1) they usually need to be operated at relatively high voltages (usually tens of volts); 2) charge carrier mobility in organic semiconductors is orders of magnitude smaller than what generally measured in their standard inorganic counterparts, putting a serious limit on the frequency range of the electrical signals that might be applied as input for organic amplifiers. A very recent attempt has been done^13^ but, as a matter of fact, the organic transistor employed in the reported experiments is always operated in the off-state and therefore, this cannot be described as an actual amplifying transducer for the cell activity.

In the following, we will present an optimized device, based on an organic thin film transistor that is able to: i) be operated at ultra-low voltages (0.5–2 V); ii) amplify signals in the frequency range of cell electrical activity (10–1000 Hz); iii) work without any external reference electrode. This important feature is possible thanks to the presence of a second gate that acts as a reference for the transistors, as further explained later on in the text. In addition, as the sensing area is separated from the active channel of the device, this new architecture allows avoiding several other drawbacks related to the measurement of signals in liquid environments by means of organic devices, such as, for instance, the poor stability of organic semiconductors when exposed to moisture and oxygen.

The basic device constituting the array, named Organic Charge-Modulated Field Effect Transistor (OCMFET), is a floating gate OTFT (Fig. 1a), whose working point is set by means of a control capacitor (Fig. 1b). A portion of the floating gate, called sensing area, on which cells are cultured, is exposed, as a direct interface, to the surrounding bio-electro-chemical environment. In particular, ionic charge variations occurring in the close proximity of the sensing area determine a charge separation in the floating gate, leading in turn to a modulation of the charge carrier density inside the channel of the transistor (Fig. 1c).

In fact, the potential of the floating gate depends on the voltage V_CG_ applied to the control gate, on the voltage V_DS_, on the capacitances present in the structure, and on the charge *Q_0_* entrapped in the floating gate due to the fabrication process, as described in the following expression:
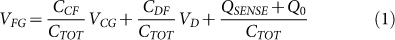
Considering all the terms as constants, apart from the charge *Q_SENSE_*, the field effect modulation can therefore be described in terms of a threshold voltage shift of the device:
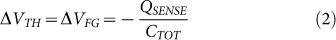
being Q_SENSE_ the charge coupled to the sensing area, and *C_TOT_ = C_CF_ +* *C_SF_* *+* *C_DF_* the sum of all the capacitances of the structure (i.e. the control capacitor *C_CF_* and the parasitic components due to the superposition between floating gate and the metal contacts lying on the opposite side of the dielectric layer, *C_SF_* and *C_DF_*). The modulation of the threshold voltage through the charge *Q_SENSE_* is the mechanism that can be employed for sensing all those chemical and biological reactions that determine a variation of charge onto or in a close proximity of the sensing area. In particular, a similar device has been successfully employed for pH sensing^14^ and as DNA hybridization sensor, both in organic^15^ and CMOS^16^ technology.

In the specific application targeted in this work, i.e. monitoring of the electrical activity of cells, the charge variation to be sensed by the device is not a quasi-static charge variation but is related to the rapid ionic displacement occurring across the cell membrane that faces the sensing area during an action potential, as previously shown in Fig. 1c. This small charge variation leads to a charge re-distribution in the floating gate, thus modulating the density of charge carriers inside the channel of the transistor. As a result, an output current variation is obtained and can be straightforward processed by a dedicated readout circuit. Being the working point of the device set by means of the control gate, the sensor does not require an additional or external reference electrode, thus overcoming one of the major drawbacks of MEAs, standard FET-based devices and standard OTFTs. This particular feature, together with the advantages given by the use of organic materials, makes this device an excellent candidate for advanced *in-vivo* bio-electronic interfaces.

A crucial requirement for successfully employing this device with living cells (especially when targeting *in-vivo* interfaces) is that the applied static voltages must be kept as small as possible to reduce risks of undesired electrochemical processes and enhance sensitivity. In order to fulfill this constraint and to optimize the cell-transistor interface for detecting rapidly varying, low amplitude extracellular electrophysiological signals, we redesigned a structure of ultralow-voltage OTFTs, similarly to what previously described in Ref. 17 (Fig. 2a). In particular, the gate dielectric was fabricated using a combination of a few nanometers of a native metal oxide layer and a 40 nm thick Parylene C film^18^. The combination of ultrathin films of metal oxide and Parylene C in a similar device structure already gave very interesting results in terms of charge sensitivity, as demonstrated in the detection of DNA single strands^15^. Another important requirement is the stability of the organic semiconductor layer. In Fig. 2b both output and input characteristics are shown for an ultralow-voltage OCMFET right after its fabrication (left) and after 10 days inside an incubator at 37°C and 95% of humidity (right). As it can be noticed, the current shows a certain degradation (as the active device area has not been encapsulated), but the transistor behavior is fully preserved. Eight identical devices, operated with a control gate voltage of −1.0 V were fabricated on a plastic substrate (Fig. 2c), having a global size of 50 × 50 mm to ensure compatibility with standard Multi Channels Systems ground plates (www.multichannelsystems.com). A glass ring delimited the sensing pads of the 8 devices, thus allowing to preserve the transistors semiconductor layers from the contact with the culture medium. Furthermore, a multichannel readout circuit was specifically designed and realized in order to perform the conditioning of the signals coming from the sensors. In Fig. 2d, an image of the OCMFET readout circuit plugged into the Multi Channel Systems ground plate is shown.

The potential of this device in cell activity monitoring is crucially correlated to its capability of detecting very small amounts of charge (less than 1 pC) in a frequency range up to 1 kHz. Therefore, in order to optimize the frequency response of the OCMFETs, we have employed a self-aligned structure^18^, that allowed obtaining a dramatic reduction of the parasitic capacitances, due to the reduction of the overlapping area between source and drain electrodes with the underneath floating gate. The ability of this device to record relatively high frequency signals is shown in Fig. 2e. The transistor is able to amplify signals at frequencies up to 1 kHz, a frequency that is higher than the typical frequency range of signals from electrogenic cells (both cardiac and neuronal cells).

Since the device has been fabricated with materials that are intrinsically biocompatible, we tested and demonstrated the biocompatibility of the whole structure. As it can be observed in Fig. 3a and 3b, cardiac cells covering the surface of the sensing electrode appear well adherent and well differentiated. Furthermore, the healthy condition of the culture was ultimately proved by the electrophysiological activity recordings made with passive metal microelectrodes (that were fabricated inside the active area delimited by the glass ring) and by Calcium imaging. A sustained and repetitive electrical activity at about 1 Hz frequency was successfully recorded (Fig. 3c) with features comparable to what is routinely obtained with standard MEAs and silicon FET devices^19^,^20^. In the Supplementary Information session, short movies are available, showing the intracellular transient of Ca^2+^ waves and the correlated cardiomyocytes contractions (Movies S1 and S2). Furthermore the biocompatibility was demonstrated by coupling neuronal cultures to our devices (see Supplementary Information fig. S1). Such networks remained in healthy conditions for more than 3 weeks, demonstrating the suitability of our system to long-term *in-vitro* applications.

In order to prove the ability of the device to record signals generated by living cells, the device was initially tested using primary cultures of cardiomyocytes from rat embryos (embryonic day 18), which develop in culture, in a few days, spontaneous and continuous mechano-electrical activity with a beat rate of 0.4 Hz–4 Hz. The OCMFET device was able to detect the occurrence of the signals (similarly to what MEA-based devices do), the beat rate and its rapid changing related to pharmacological modulations, obtained by applying both positive and negative chronotropic compounds^21^,^22^. In Fig. 4a and 4b examples of extracellular action potentials measured, respectively, with an OCMFET and a metal microelectrode fabricated on the same chip, are shown. Considering a p-type organic transistor and a first inverting stage of the readout electronics, the shape of the single signal shown in Fig. 4a is consistent with the expected current fluctuation caused by the ionic charge displacement in the early phase of the cardiac action potential. The *I_DS_* fluctuation is driven by the fast movement of Na^+^ ions occurring at the very beginning of the intracellular action potential (Fig. 4c). The fast entrance of Na^+^ inside the cell (phase 0) induce a temporary (0.5–2 ms) displacement of positive ions rapidly entering in the portion of the cell membrane that faces the sensing area. Due to the capacitive coupling between the cells and the sensing area, a displacement charge is induced into the floating gate area facing the cells; this small and transient amount of charge induces a negative charge increase in the area underneath the transistor channel and this leads to an increase of the channel current (as previously shown in Fig. 2c). The subsequent movement of K^+^ ions from the inside to the outside of the cell (phase 1) is responsible for the temporary (0.5–2 ms) displacement of charge in the floating gate in the opposite direction, thus causing a decrease of the channel current. As it can be noticed by comparing Fig. 4a and 4b, the shape of the extracellular measured signal is similar in the two cases demonstrating the detection capabilities of the reference-less OCMFET device. Indeed, such inflow and outflow of ions do not influence the concentration of sodium (and could slightly affect potassium) as the displacement of ions during an action potential is negligible with respect to the mM concentration in the extracellular medium. However, these small charge displacements, thanks to the high capacitive coupling and high sealing resistance^23^ determine the detectability of the signals with a mechanism somehow similar as the one previously reported for silicon based Insulated FET^5^.

To extensively test our system, we then performed systematic measurements by recording the spontaneous electrophysiological activity of rat cardiomyocytes (8 Days InVitro - DIV) by means of the OCMFET device array (Fig. 5a). To further confirm the recording capabilities of the developed OCMFET based system, we modulated the cell activity by slowly varying the temperature of the culture's medium. We simply changed the control temperature of the thermostat, in contact with the cell plate, from 35°C to 40°C and back. As expected, and shown in Fig. 5b, the culture's beating frequency varied consistently with the temperature variations. Finally, to ultimately prove that the recorded signals were generated by the cardiomyocyte cells, after several recordings made in physiological conditions (i.e. at 37°C in a standard culture medium and a controlled atmosphere), the spontaneous activity of the cardiomyocytes' culture was pharmacologically manipulated by administering 100 μM of Norepinephrine (a specific cardio-stimulant that acts on β-adrenergic receptors^21^), and then a high-dose (100 μM) of Verapamil (a calcium blocker specific to the L-type calcium channel that acts as a cardio-relaxant^22^). As expected, the OCMFET recordings showed modulations of cellular activity as reported in Fig. 5c. All the measurements relative to the pharmacological modulation of the electrical activity were performed while maintaining the culture at constant temperature (37°C) and the same reliable results were obtained in 5 recording devices simultaneously. With this array configuration, it was possible to estimate the propagation speed of the electrical signal (around 0.4 m/s, in agreement with values measured by means of other techniques^24^). Figure 5d shows the raster plot of the propagation of the action potential among the cell monolayer, as recorded by different OCMFET devices fabricated onto the same substrate.

Measurements performed with several OCMFETs showed *I_DS_* variations ranging from hundreds of pA to few nA. Considering an average *I_DS_* variation of 1 nA and representative values of the electronic parameters of the device (a trans-conductance *g_m_* of 300 pA/mV and the sum of the capacitances *C_TOT_* of 100 pF, see Supplementary Information, Table S1), we were able to estimate, using the OCMFET's equations, a corresponding charge variation of ~0.3 pC. By assuming that this variation is entirely due to the ionic charge crossing the cell membrane during the upstroke of an action potential and by considering a typical membrane capacitance of 1 μF/cm^2^ 25, we may compute a value of about 300 μm^2^ for the effective area where this charge variation occurs. This value is consistent with the typical adhesion area of the cardiomyocyte soma, thus indicating the validity of the transduction principle. To test the suitability of our system for future applications with neuronal cells, the device was also tested with rat striatal cells. Figure 5e shows a preliminary recording of the electrical activity of a culture of rat striatal neurons maintained in vitro for 21 days (21 DIV). A picture of the neurons cultured on the same plastic platform together with the comparison with a signal recorded by a standard MEA is also available in the Supplementary Information Session. Interestingly, calculation of the maximal amplitude of the signal-to-noise ratio^26^ provided a value up to 3.2 (similarly to what is routinely obtained with standard MEAs)

In summary, the reliable detection of action potentials (both in physiological conditions and upon stimulation obtained by heating/cooling steps and by pharmacological manipulations) from cardiac cells, and the consistency of the beating frequency with that recorded with standard microelectrodes fabricated onto the same substrates, demonstrates the capability of the reference-less OTFT-based device to efficiently transduce electrophysiological signals from electrogenic cells. The suitability of the proposed system for future applications with neurons was also preliminary successfully tested with rat striatal neurons.

Moreover, a simple working mechanism based on the capacitive coupling between the floating gate of the transistor and the cellular membrane crossed by the ionic charge during the action potential, justifies the observed signal shape and amplitude.

The obtained results support the use of this device for the detection of the electrical activity of any kind of excitable and spontaneously active cells and suggest new applications in the neuro-electronic interface domain. The features of the presented platform could be further exploited for realizing smart, disposable substrates for cell cultures at low cost, thus opening an innovative perspective for functional cell monitoring both *in-vitro* and *in-vivo*.

## Methods

### Sensor fabrication

All devices were fabricated on a 175 μm thick polyethylene terephthalate (PET) substrate (Goodfellow). At first, a metallic layer (Al or Ti, Goodfellow) was thermally evaporated onto the substrate and patterned by means of a standard photolithographic process. This patterned metallic film act as the floating gates in the final sensor architecture. In particular, the terminal sensing pads of the floating gates have a diameter ranging from 50 to 150 μm. The microelectrodes were patterned during this step of the fabrication process. An UV-Ozone treatment was subsequently performed in order to enhance the growth of the superficial native oxide layer. After that, 30–50 nm of Parylene C (Specialty Coating Systems) were deposited onto the entire substrate. A gold layer was then evaporated and patterned using a self-alignment process as described in Ref. 18 to obtain the drain, source, and control gate contacts. The obtained self-aligned low-voltage OTFTs have a W/L (channel Width/channels Length) ratio of about 650. A thick layer of photoresist was finally deposited and photo-lithographed in order to expose only a portion of the pads and protect the rest of the surface. Afterwards, the Parylene C layer was etched from both the floating gates pads and the microelectrodes by means of a plasma oxygen exposure^27^. After the photoresist removal, 2 μl of a solution of 6,13-Bis(triisopropylsilylethynyl)pentacene (TIPS Pentacene, Sigma Aldrich) in toluene (0.5 wt %) were drop casted directly over the channel of the transistors. Finally, a glass ring (1.5 cm in diameter) was glued onto the substrate with a thin rim of polydimethylsiloxane (PDMS) in order to delimit the cell culture region. A careful cleaning of the chip surface with ethanol, acetone and deionized water preceded every step of the process.

### Experimental setup

The experimental setup consisted of a multichannel (16 channels) dedicated readout and conditioning electronics. Each channel consists of three main blocks: a first inverting I/V converter with a 1 MΩ feedback resistor, a 2^nd^ order high pass Butterworth filter with a cut-off frequency of 150 Hz, a 3^rd^ order low pass Butterworth filter with a cut-off frequency of 1.3 kHz; all channels share an adjustable biasing circuit for the OCMFETs polarization (V_DS_ = V_GS_ = −1 V for all the reported measurements). The total voltage gain of the circuit is 110. The realized custom circuit is connected to a Multichannel Systems acquisition board for A/D conversion, acquisition and storage (www.multichannelsystems.com). The measurements with the passive microelectrodes were performed by simply substituting the OCMFET readout electronics with a MEA1060-inv amplifier (www.multichannelsystems.com) provided with the necessary external reference electrode. All the measurement sessions were carried out inside a Faraday cage in order to minimize the electrical environmental noise on the system.

## Author Contributions

A.S. made the entire experimental work, from the design and fabrication of the devices to the implementation of the measurement setup for the electrophysiological measurements. He also contributed to write the paper. S.L. contributed to the design and discussions about the working models of the device. He also contributed to write the paper. P.C. contributed to the fabrication procedure and to the discussion of the results. He also contributed to write the paper. M.T. took entirely care of the preparation of cell cultures and contributed to the experiments with living cells. She also contributed to write the paper. S.M. coordinated the work, focusing in particular on the design of the measurement platform and on the electrophysiological experiments. He also contributed to write the paper. A.B. coordinated the work, focusing in particular on the design and fabrication of the device and working platform. She coordinated the writing of the paper.

## Supplementary Material

Supplementary Information Supplementary Material

Supplementary Information Movie 1

Supplementary Information Movie 2

## Figures and Tables

**Figure 1:**
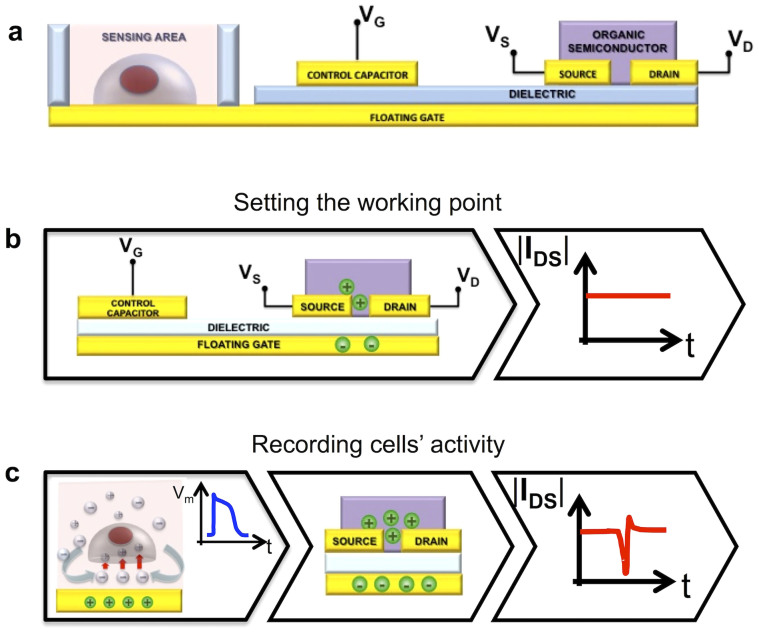
Working principle of the OCMFET. (a), Cross section of an OCMFET device. (b), The working point is set by applying appropriate V_GS_ and V_DS_. (c), The charge fluctuation over the sensing area determines a charge re-distribution inside the floating gate, which modulates the charge carriers density inside the channel of the transistor. As a result, a variation of the output current is obtained.

**Figure 2:**
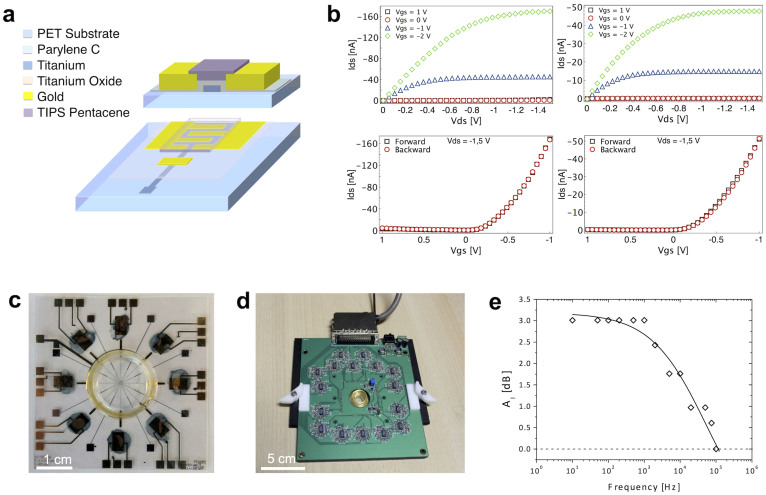
Structure and electrical characteristics of the device. (a), Schematic structure of the material layers composing the device. (b), Typical characteristics of an ultra-low voltage OCMFET right after the fabrication (left) and after 10 days inside an incubator (right). (c), A complete device with 8 OCMFETs and 8 microelectrodes; in the center of the substrate, the sensing pads are surrounded by a glass ring that allows confining the culture medium with the cells. (d), Top view of the complete experimental setup. The readout electronics in inserted onto a Multichannel Systems ground plate. (e), Gain vs frequency relationship for an OCMFET device employed for the measurements with cells.

**Figure 3:**
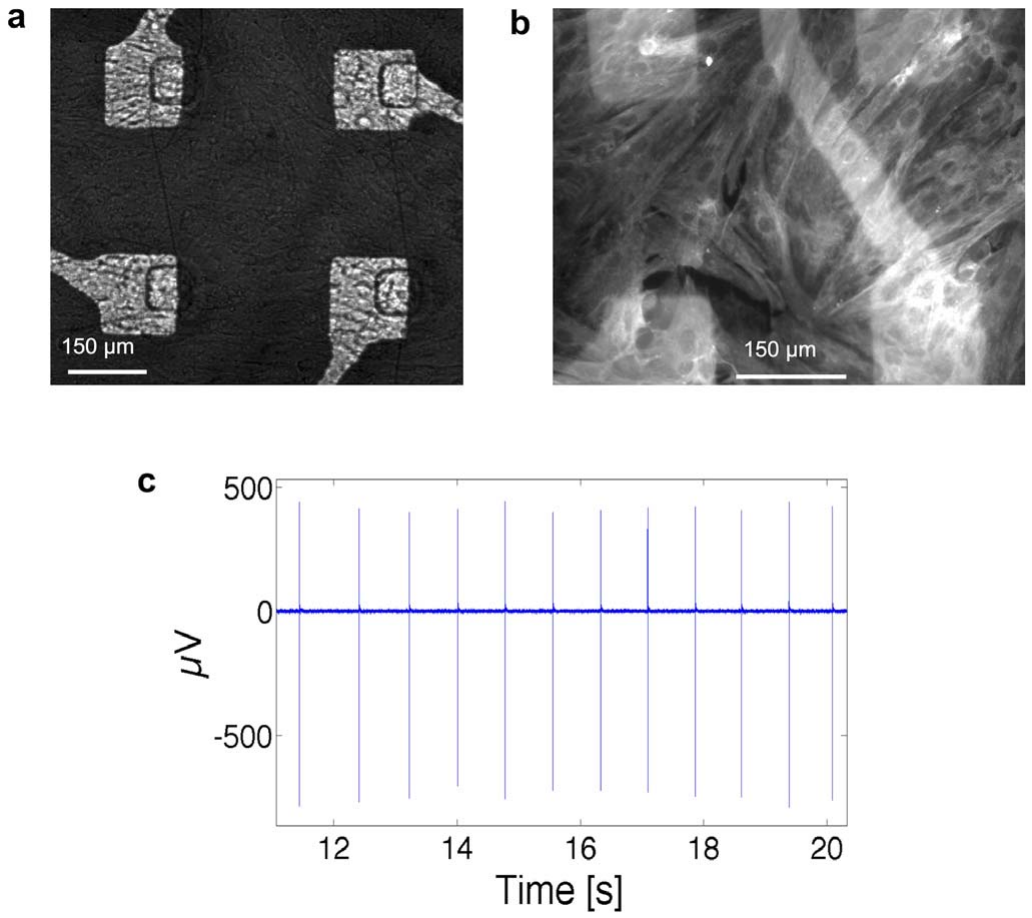
Biocompatibility assessment. (a), Magnification of the sensing area: a confluent culture of rat cardiomyocytes (8 DIV) is adhering on the surface. (b), Cardiomyocytes culture fixed after a recording session and immunostained for the sarcomeric protein Tropomyosin. (c), Few seconds of spontaneous activity measured with a metallic microelectrode fabricated near a floating gate pad and recorded with a MEA1060-Inv Multichannel Systems amplifier.

**Figure 4:**
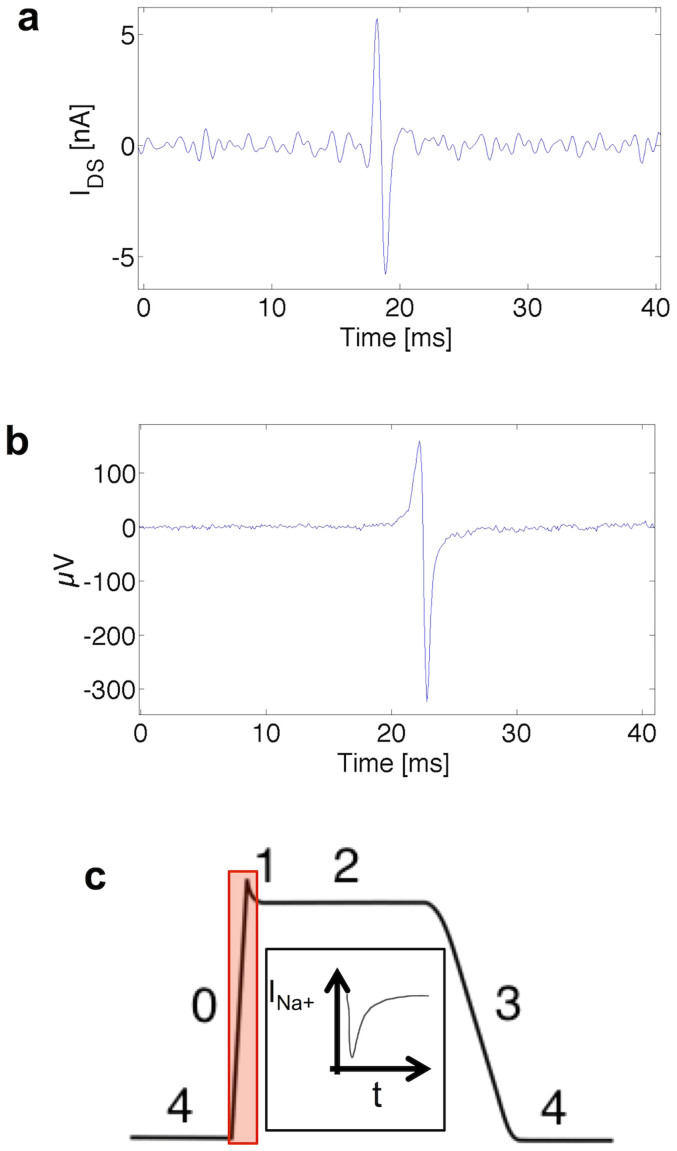
Comparison between OFET signals, MEA extracellular APs and intracellular ventricular APs. (a), Single signal measured with an OCMFET. The fast ionic displacement during the early phase of the intracellular cardiac AP elicits an increase followed by a decrease of the current of the OTFT. The shape and the duration are coherent with the sensing of the events occurring during the upstroke of the intracellular AP. (b), Rat cardiomyocyte extracellular AP measured with a passive microelectrode. It is worth noting the similarity of the capacitive part of the MEA signal and the OFET signal. (c), Cardiac intracellular action potential; in red the initial part corresponding to the rapid entry of Na^+^ ions inside the cell. (c) (**inset**), A representation of the Na^+^ current, the fast current responsible for the action potential upstroke.

**Figure 5:**
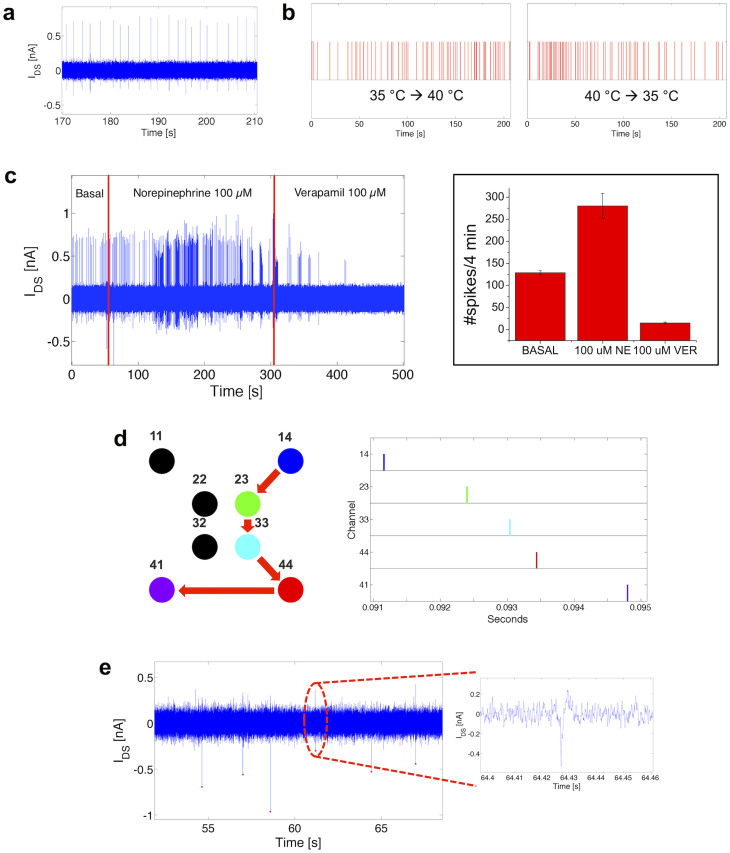
Experimental results. (a), Spontaneous activity of a rat cardiomyocytes culture (8 DIV) mantained at 37°C measured with an OCMFET. (b), Raster plots representing the thermal modulation of the spontaneous activity of the culture. The temperature was incremented from 35°C to 40°C (left plot) and decreased from 40°C to 35°C (right plot) causing a consistent variation of the beating frequency. (c), Chemical tuning of the culture's activity. The spontaneous activity was accelerated by means of the addition of 100 μM of Norepinephrine and then suppressed with 100 μM of Verapamil. (c) (**inset**), Beating frequency modulation (statistics on 5 OCMFETs - average and standard deviation): spike-count on 4 min of basal (129 ± 4.6), NE-mediated (280 ± 28.6) and VER-mediated activity (15 ± 1.9). (d), Signal propagation reconstruction in a device with multiple recording sites: colored and black dots represents, respectively, OCMFETs that recorded signals and “silent” OCMFETs (left); raster plot of the spontaneous activity of the “colored” channels indicating a propagation of the signal from site 14 to site 41 (right). (e), Action potentials of striatal cells from rat embryo (21 DIV) mantained at 37°C measured with an OCMFET. (e) (**inset**), Particular of a neuronal extracellular action potential.

## References

[b1] Thomas C. A. Jr, Springer P. A., Loeb G. E., Berwald-Netter Y. & Okun L. M. A miniature microelectrode array to monitor the bioelectric activity of cultured cells. Exp. Cell Res. 74, 61–66 (1972). 4672477 10.1016/0014-4827(72)90481-8

[b2] Grattarola M. & Martinoia S. Modeling the Neuron-Microtransducer Junction: From Extracellular to Patch Recording. IEEE Trans. Bio-medical Engineering 40, 158–165 (1993). 10.1109/10.204769 8468074

[b3] Bergveld P. Development, Operation, and Application of the Tool for Electrophysiology. IEEE Trans. Bio. Engineering 19, 342–351 (1972). 10.1109/TBME.1972.324137 5038390

[b4] Bergveld P., Wiersma J. & Meertens H. Extracellular Potential Recordings by Means of a Field Effect Transistor Without Gate Metal, Called OSFET. IEEE Trans. Bio. Engineering 23, 136–144 (1976). 10.1109/tbme.1976.324574 1248839

[b5] Fromherz P. & Offenhausser A. A Neuron-Silicon Junction: A Retzius Cell of the Leech on an Insulated-Gate Field-Effect Transistor. Science 252, 1290–1293 (1991). 1925540 10.1126/science.1925540

[b6] Martinoia S. *et al.* Development of ISFET array-based microsystems for bioelectrochemical measurements of cell populations. Biosensors & bioelectronics 16, 1043–1050 (2001). 11679287 10.1016/s0956-5663(01)00202-0

[b7] Berggren M. & Richter-Dahlfors A. Organic bioelectronics. Adv. Mat. 19, 3201–3213 (2007).

[b8] Simon D. T. *et al.* Organic electronics for precise delivery of neurotransmitters to modulate mammalian sensory function. Nature Mater. 8, 742–746 (2009). 19578335 10.1038/nmat2494

[b9] Ghezzi D. *et al.* A polymer optoelectronic interface restores light sensitivity in blind rat retinas. Nature Photonics 7, 400–406 (2013). 10.1038/nphoton.2013.34 PMC4855023 27158258

[b10] Blau A. *et al.* Flexible, all-polymer microelectrode arrays for the capture of cardiac and neuronal signals. Biomaterials 32, 1778–1786 (2011). 21145588 10.1016/j.biomaterials.2010.11.014

[b11] Bernards D. A. & Malliaras G. G. Steady-State and Transient Behavior of Organic Electrochemical Transistors. Adv. Fun. Mater. 17, 3538–3544 (2007).

[b12] Khodagholy D. *et al.* In vivo recordings of brain activity using organic transistors. Nature Comm. 4, 1575–1579 (2013). 10.1038/ncomms2573 PMC3615373 23481383

[b13] Benfenati V. *et al.* A transparent organic transistor structure for bidirectional stimulation and recording of primary neurons. Nature Mater. 12, 672–680 (2013). 23644524 10.1038/nmat3630

[b14] Caboni A., Orgiu E., Scavetta E., Barbaro M. & Bonfiglio A. Organic-based sensor for chemical detection in aqueous solution. Appl. Phys. Lett. 95, http://dx.doi.org/10.1063/1.3232252 (2009).

[b15] Lai S. *et al.* Ultralow voltage, OTFT-based sensor for label-free DNA detection. Adv. Mater. 25, 103–107 (2013). 23027594 10.1002/adma.201202996

[b16] Barbaro M., Bonfiglio A. & Raffo L. A charge-modulated FET for detection of biomolecular processes: conception, modeling, and simulation. IEEE Trans. El. Dev. 53, 158–166 (2006).

[b17] Cosseddu P., Lai S., Barbaro M. & Bonfiglio A. Ultra-low voltage, organic thin film transistors fabricated on plastic substrates by a highly reproducible process. Appl. Phys. Lett. 100, http://dx.doi.org/10.1063/1.3691181 (2012).

[b18] Lai S., Cosseddu P., Gazzadi G. C., Barbaro M. & Bonfiglio A. Towards high frequency performances of ultra-low voltage OTFTs: Combining self-alignment and hybrid, nanosized dielectrics. Organic Electronics 14, 754–761 (2013).

[b19] Denyer M. C. T., Riehle M., Britland S. T. & Offenhausser A. Preliminary study on the suitability of a pharmacological bio-assay based on cardiac myocytes cultured over microfabricated microelectrode arrays. Med. Biol. Eng. Comput. 36, 638–644 (1998). 10367451 10.1007/BF02524437

[b20] Hecken H. *et al.* 64-Channel extended gate electrode arrays for extracellular signal recording. Electrochimica Acta 48, 3355–3362 (2003).

[b21] Simpson P. Stimulation of hypertrophy of cultured neonatal rat heart cells through an alpha 1-adrenergic receptor and induction of beating through an alpha 1- and beta 1-adrenergic receptor interaction. Evidence for independent regulation of growth and beating. Circ. Res. 56, 884–894 (1985). 2988814 10.1161/01.res.56.6.884

[b22] Yonemochi H., Saikawa T., Takakura T., Ito S. & Takaki R. Effects of calcium antagonist on β-receptors of cultured cardiac myocytes isolated from neonatal rat ventricle. Circulation 81, 1401–1408 (1990). 2156639 10.1161/01.cir.81.4.1401

[b23] Martinoia S. & Massobrio P. ISFET–neuron junction: circuit models and extracellular signal simulations. Biosensors and Bioelectronics 19, 1487–1496 (2004). 15093221 10.1016/j.bios.2003.12.003

[b24] Darrow B. J., Fast V. G., Kléber A. G., Beyer E. C. & Saffitz J. E. Increased Conduction Velocity and Enhanced Connexin Expression in Dibutyryl cAMP–Treated Cultured Cardiac Myocytes. Circ. Res. 79, 174–183 (1996). 8755993 10.1161/01.res.79.2.174

[b25] Ingebrandt S., Yeung C., Krause M. & Offenhausser A. Cardiomyocyte-transistor-hybrids for sensor application. Biosensors Bioelectronics 16, 565–570 (2001). 11544050 10.1016/s0956-5663(01)00170-1

[b26] Maccione A. *et al.* A novel algorithm for precise identification of spikes in extracellularly recorded neuronal signals. J. Neurosci. Methods 177, 241–249 (2009). 18957306 10.1016/j.jneumeth.2008.09.026

[b27] Trantidou T., Prodromakis T. & Toumazou C. Oxygen plasma induced hydrophilicity of Parylene-C thin films. Appl. Surf. Sci. 261, 43–51 (2012).

