# Supplementary material for: An organic transistor-based system for reference-less electrophysiological monitoring of excitable cells
Source: Sci Rep. 2015 Mar 6;5:8807. doi: 10.1038/srep08807 (PMC4351515; doi:10.1038/srep08807)
Supplement: Supplementary Information — Supplementary Material [file srep08807-s1.pdf]

Title: An organic transistor-based system for reference-less electrophysiological monitoring of excitable cells

Authors: **A. Spanu[1,2], S. Lai[1], P. Cosseddu[1], M. Tedesco[2], S. Martinoia[2]*, and A.Bonfiglio[1,3]***

Supplementary Material.

**Mobility [$cm^2/V*s$]**	**V_{TH} [V]**	**g_m [A/V]**	**I_{ON}/I_{OFF}**
$(1.4 \pm 0.6)10^{-2}$	0.20 ± 0.40	$(3.0 \pm 0.7)10^{-7}$	$(2.6 \pm 1.7)10^{3}$

Table S 1. Statistics on the characteristic parameters of 11 identical OCMFETs (average and standard deviation). The g_m is calculated in the saturation region for V_{DS} = -1 V.

Cell cultures

Embryonic hearts were micro-dissected away from 18 days rat embryos and then plated and grown on the floating gate surface of the OCMFET devices. Briefly, after the hearts were isolated we transferred them to an ice-cold buffer solution containing 100 mM NaCl, 10 mM KCl, 1.2 mM KH_2PO_4, 4 mM $MgSO_4$, 50 mM Taurina, 20 mM Glucose, 10 mM Hepes, pH 7.0 (*1*). The atria and vascular tissues were removed and the ventricles minced into small pieces of 1-3 mm^3. Ventricular tissue was enzymatically digested in 0.2% Collagenase Type II (cod. 17101 Invitrogen) and 0.4% Pancreatin (P-3292 Sigma) diluted in the same buffer solution above mentioned. Short repetitive cycles of digestion help to obtain a good yield of cell viability, therefore the tissue fragments were incubated a 37°C for 10'-15'. Cell suspension, originated from each cycle of digestion, was transferred into a conical centrifuge tube with DMEM-Glutamax (31966 Invitrogen) 10% FBS (Sigma) solution and was centrifuged for 8' at 1200 rpm.

The resulting pellets from each digested suspension were pooled and then re-suspended in culture medium consisting in (4:1) DMEM-Glutamax /M199 (M7653 Sigma), 6% HS, 4% FBS, 10 μg/ml Gentamycin. This cell suspension was pre-plated on petri dishes and incubated a 37°C in 95% humidity 5% CO_2 for 2 h. During this period the non-myocyte cell types, predominantly fibroblast cells, adhered to the culture dish but the cardiomyocytes (which have slower time of adhesion) remained in suspension. After incubation, the medium, containing predominantly cardiomyocyte cells, was removed and gently centrifuged. Cardiomyocytes enriched population was resuspended in culture medium and finally plated onto the sensing area of the devices at $5*10^4$ cells per well. The procedure was approved by the European Animal Care Legislation and by the guidelines of the University of Genova.

One day before the culture preparation, the surface of the OCMFET was sterilized with Ethanol 70°, exposed to Plasma Oxigen (30 W for 30 seconds) to improve the hydrophilicity of the Parylene C layer and coated with laminin solution (50 μg/ml L-2020 Sigma). OCMFETs were placed at 37 °C in the incubator until the step of the dissection. On the day of cell collection the laminin solution was removed and the cell culture surface (i.e., the area within the glass ring) was washed twice with sterile water and left to dry on the laminar hood. The embryonic cardiomyocyte cells were then plated at the final number of $5 \cdot 10^4$ cells per well and spontaneous beating was obtained within the first 24 h. During the first three days of culture, cells formed a confluent layer and synchronized their beating throughout the entire culture.

Immunofluorescent detection for tropomyosin and F-actin was applied to highlight myofibrils in our cultures. The cells were fixed in 4% Formaldehyde and pemeabilized with 0.1% Triton-X100. Monoclonal antibody Anti-Tropomyosin 1:400 (Sigma T2780), with II° antibody goat anti mouse Alexa Fluor 546 (1:1000) and Phalloidin Alexa-Fluor488 conjugated

1:200 (Lonza PA-3010) for F-Actin were diluted in PBS plus 2% BSA, 0.5 % FBS at pH 7.4 staining solution.

In order to isolate and culture the fetal striatal neurons, rat embryos at day 18 were anesthetized by exposure to CO_2 and sacrificed. Heads were collected in Ca^{2+}/Mg^{2+}-free Hank's Balance Salt Solution with 10 mM HEPES, CMF-HBSS, the same solution in which the whole dissection took place. Briefly, the brain was removed from the skull and the ganglionic eminence regions were isolated from the single hemispheres, which were positioned with the ventral aspect facing up. Striatal tissue was transferred in a sterile conical tubes where it was dissociated in 0.125% of Trypsin/Hank's solution containing 0.05% of DNAse (D-5025 Sigma-Aldrich) for 15-18 min at 37 °C. The supernatant solution was removed and the enzymatic digestion was stopped by adding 10% fetal bovine serum (FBS) in Neurobasal medium for 5 min. Medium with FBS was removed and replaced with culture medium Neurobasal supplemented with B27, 1% Glutamax, gentamicin 10 µg/ml (Gibco Invitrogen). Cells were plated at a total density of $1*10^5$ cells in each device. A sterilization of the OCMFET was also performed and to increase the wettability and to improve the neurons' surface adhesion onto the sensing active area, we followed again the same procedures that we have described above. The cultures, containing both glia and neurons, were incubated at 37 °C in a humidified 5% CO_2 incubator, after initial plating, half of the medium was exchanged with fresh medium every 3-4 days (*2*).

References

1. Rapila, R., Korhonen, T., Tavi, P. Excitation–contraction coupling of the mouse embryonic cardiomyocyte. *J. Gen. Physiol.* **132,** 397-405 (2008).

2. Banker, G., Goslin, K. *Culturing Nerve Cells 2nd Edition*. (MIT Press, 1998).

Movie S 1. Calcium imaging of rat cardiomyocytes cultured onto a OCMFET device. The cardiomyocytes culture (8 DIV) was loaded with 3µM (final concentration), of Oregon Green-Bapta1-AM (Invitrogen-Molecular Probes). The movie was acquired at 20X magnification using a BX51 Olympus upright microscope and an Hamamatsu Orca-ERII CCD Camera.

Movie S 2. Spontaneous activity of rat cardiomyocytes cultured onto an OCMFET device. The movie was acquired at 10X magnification using a BX51 Olympus DIC upright microscope and an Hamamatsu Orca-ERII CCD Camera.

Figure S 1. Recordings of the spontaneous activity of rat embryo striatal neurons. a, Rat embryo striatal neurons (21 DIV) cultured onto an OCMFET device. The image was acquired a BX51 Olympus DIC upright microscope. **b**, Activity of the striatal neurons culture measured with an OCMFET. **c,** Extracellular activity of a culture of rat embryo striatal neurons recorded with a 60MEA100/10iR-Ti and a MEA1060-Inv Multichannel Systems amplifier. It is worth noting that the frequency, the amplitude, and the shape of the signals is comparable for both the recordings.

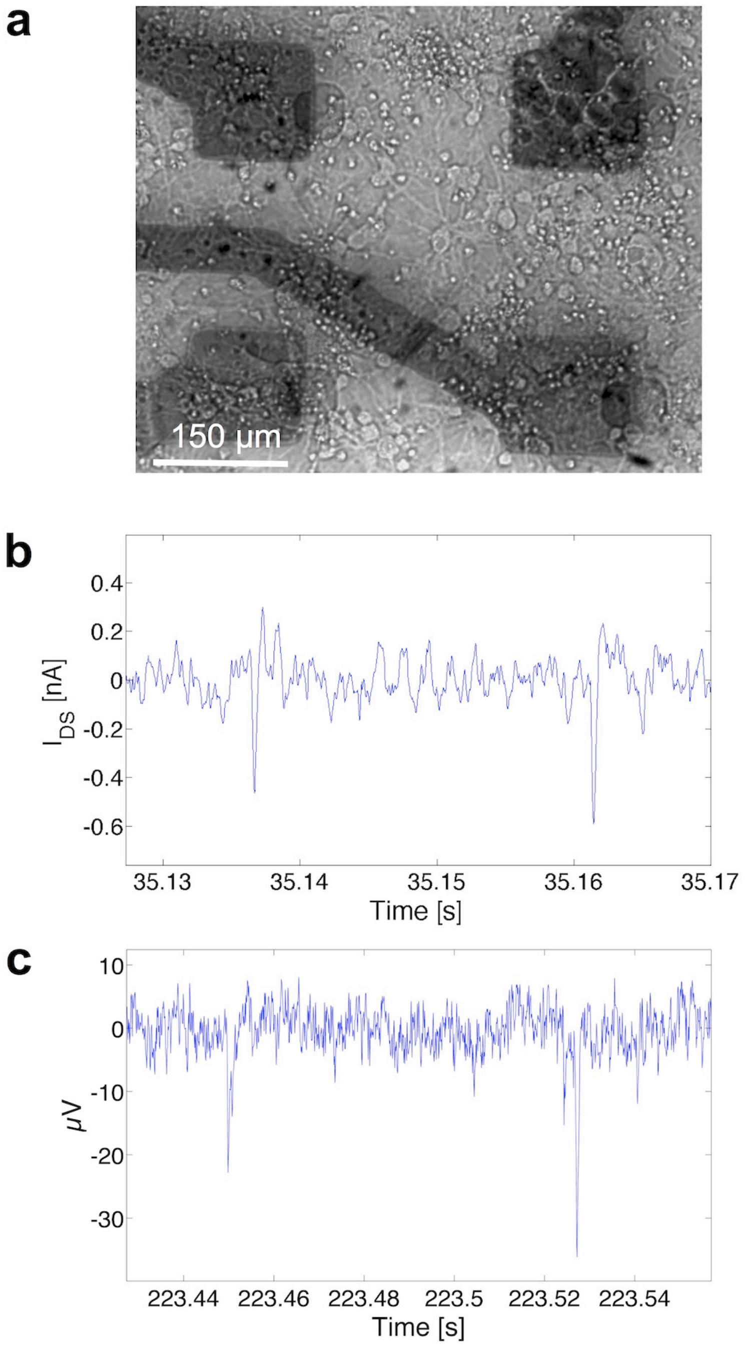
a
150 μm
b
0.4
0.2
0
-0.2
-0.4
-0.6
I_DS [nA]
35.13
35.14
35.15
35.16
35.17
Time [s]
c
10
0
-10
-20
-30
μV
223.44
223.46
223.48
223.5
223.52
223.54
Time [s]

Figure S 1
